# External Lumbar Drainage for Refractory Intracranial Hypertension in Traumatic Brain Injury: A Systematic Review

**DOI:** 10.7759/cureus.30033

**Published:** 2022-10-07

**Authors:** Andrew R Stevens, Wai C Soon, Yasir A Chowdhury, Emma Toman, Sebastian Yim, Tonny Veenith, Ramesh Chelvarajah, Antonio Belli, David Davies

**Affiliations:** 1 Neurosurgery, University Hospitals Birmingham, Birmingham, GBR; 2 National Institute for Health Research Surgical Reconstruction and Microbiology Research Centre, University Hospitals Birmingham, Birmingham, GBR; 3 Institute of Inflammation and Ageing, University of Birmingham, Birmingham, GBR; 4 Medical and Dental Sciences, University of Birmingham, Birmingham, GBR; 5 Anaesthesia and Critical Care, University Hospitals Birmingham, Birmingham, GBR; 6 Life and Environmental Sciences, University of Birmingham, Birmingham, GBR; 7 Centre for Trauma Sciences Research, University of Birmingham, Birmingham, GBR

**Keywords:** neurosurgery, critical care, cerebrospinal fluid drainage, intracranial hypertension, traumatic brain injury

## Abstract

Considerable variation exists in the clinical practice of cerebrospinal fluid diversion for medically refractory intracranial hypertension in patients with acute traumatic brain injury (TBI), which is achievable via lumbar or ventricular drainage. This systematic review sought to compile the available evidence for the efficacy and safety of the use of lumbar drains for intracranial pressure (ICP) control.

A systematic review of the literature was performed with the search and data extraction performed by two reviewers independently in duplicate. Nine independent studies were identified, enrolling 230 patients, 159 with TBI. Efficacy for ICP control was observed across all studies, with immediate and sustained effect, reducing medical therapy requirements. Lumbar drainage with medical therapy appears effective when used alone and as an adjunct to ventricular drainage. Safety reporting varied in quality. Clinical or radiological incidents of cerebral herniation (with an unclear relationship to lumbar drainage) were observed in 14/230 patients resulting in one incident of morbidity without adverse patient outcome.

The available data is generally poor in quality and volume, but supportive of the efficacy of lumbar drainage for ICP control. Few reports of adverse outcomes are suggestive of, but are insufficient to confirm, the safety of use in the appropriate patient and clinical setting. Further large prospective observational studies are required to generate sufficient support of an acceptable safety profile.

## Introduction and background

Established management practices for moderate and severe traumatic brain injury (TBI) are centred on minimising secondary injury through normalising intracranial homeostasis. This is achieved through a therapeutic paradigm principally centred on the avoidance of raised intracranial pressure (ICP) and maintenance of cerebral perfusion pressure (CPP). Contemporary ICP management strategies utilise a sequential escalation of therapeutic intensity until ICP control is achieved, with protocols based on the Brain Trauma Foundation (BTF) guidelines [1]. Initial medical treatments for intracranial hypertension include sedation, mild hypocapnia, and hyperosmolar therapy. Where intracranial hypertension is refractory to these interventions, therapies including diversion of cerebrospinal fluid (CSF), barbiturate coma, and decompressive craniectomy can be considered.

CSF diversion to manage raised ICP is classically understood in terms of the Monro-Kellie doctrine [2,3]. The principle states that diversion (or buffering) of one constituent of the intracranial compartment (e.g., CSF) permits an increasing volume of other constituents (i.e., parenchymal oedema in response to trauma). CSF can be removed from the system through two principal access points: (1) a ventriculostomy in the lateral ventricle connected to an external ventricular drain (EVD); or (2) a lumbar catheter connected to an external lumbar drain (ELD). CSF drainage can be achieved intermittently through discrete episodes of volume-controlled drainage or continuously by permitting uninterrupted pressure-controlled drainage [4].

Traditionally, CSF diversion in TBI is achieved through a ventriculostomy. Siting a ventriculostomy also permits measurement of intraventricular pressure by transduction of a closed drain, though in recent years, ICP monitoring has more commonly been achieved through intraparenchymal monitoring via a transcranial access device or “bolt” [5]. Practice varies from centre to centre [6], and between adults and paediatrics, with the usage of EVD monitoring and drainage being a more common practice in paediatric trauma centres [7] but less so in adults [8].

ELD originated in 1963 as a means for reducing cerebral tension intraoperatively [9] and has become an established method of CSF diversion in a variety of settings [10], including post-traumatic CSF leak [11,12], normal pressure hydrocephalus assessment [13], skull base surgery [14,15], and in thoracoabdominal aortic surgery to reduce spinal cord ischaemia [16]. The use of ELD for ICP control after TBI is less common due to the potential risk of iatrogenic transtentorial herniation, recognised in historical examples of herniation after lumbar puncture in patients with raised ICP [17-20]. In other contexts of intracranial hypertension, the use of ELD has been successfully adopted, including in bacterial and cryptococcal meningitis [21,22] and subarachnoid haemorrhage (SAH) [23-25]; achieving similar ICP control to ventriculostomy drainage without significant complication rates, though consensus guidelines have found insufficient evidence to support ELD as an option in ICP control after TBI [26].

Rationale

Lumbar drainage represents a potential alternative means of CSF diversion to ventriculostomy due to avoidance of the passage of a drain through cerebral parenchyma. Insertion of a lumbar drain can be a technically and logistically simpler procedure, particularly in patients with isolated TBI with small lateral ventricles rendering insertion of EVD challenging or not achievable. Whilst thought to also achieve ICP control, concerns regarding the safety of lumbar drainage lie in the possible complication of cerebral herniation with associated morbidity and mortality. As such, to inform current practice and future research directions, there is a need for a summary of the safety and efficacy of external lumbar drainage for ICP control in TBI, particular with reference to the safety and efficacy of the other available CSF diversion modality, external ventricular drainage.

Objectives

Our objective is to systematically evaluate the available literature, examining the use of external lumbar drainage of CSF for refractory intracranial hypertension in traumatic brain injury (TBI). Specifically, we seek to summarise the available evidence for (1) the efficacy of the use of external lumbar drains in the control of refractory intracranial hypertension in TBI; and (2) the safety of external lumbar drainage in acute TBI.

## Review

Methods

A systematic review of the literature was performed following the methodology of the Cochrane Handbook for Systematic Reviewers and presented in accordance with the Preferred Reporting Items for Systematic Reviews and Meta-analyses (PRISMA) [27]. The review was registered with PROSPERO under registration number CRD42020192283. No amendments were made to the information provided at registration or in the protocol. This article was previously posted to the ResearchSquare pre-print server on August 2nd 2021.

The primary review questions determined by the authors were: (1) Is CSF diversion via an external lumbar drain a safe procedure in TBI?; (2) Does CSF diversion via an external lumbar drain effectively control intracranial hypertension in TBI?

Secondary review questions determined by the authors were: (3) What selection/exclusion criteria are utilised for patient selection to determine appropriate candidates for external lumbar drainage?; (4) Are there any established protocols for the use of external lumbar drainage for the control of ICP in TBI?

Inclusion Criteria

Population: The population of interest were human subjects with moderate or severe TBI, inclusive of both adult and paediatric populations.

Intervention: The intervention considered is CSF diversion via an ELD.

Outcome measures: The outcome measures of interest were ICP control, CPP optimisation and the safety profile of the intervention. A considered secondary outcome measure was any appropriate measure of functional outcome post-injury. Acceptable indices of ICP/CPP control considered are (1) direct measurement pre- and post- drainage, and (2) indirect outcome measures of reduced incidence of requiring osmotic therapy, hyperventilation, or more invasive surgical procedures (external ventricular drainage or decompressive craniectomy). For each outcome, any appropriate effect measure was accepted for the presentation of results; due to heterogeneity, no synthesis was performed and as such, no procedures were required for data conversion handling of missing summary statistics.

Setting: Given the intensive monitoring required to gain the above data, such studies would be in the intensive care unit or neurosciences critical care settings.

Methodology: Any research methodology in humans was considered for inclusion, including observational studies and case series/reports.

Exclusion Criteria

Studies conducted in animal models or in vitro human models were excluded. Studies which recorded the variables of interest, but did not report sufficient data on these variables for our research questions were also excluded.

Information Sources

We systematically searched the following database from their respective inception to September 2020: Medline, Embase, Cochrane Central, NICE Evidence, Google Scholar and Web of Science. Reference lists of pertinent review articles on the topic were hand-searched for suitable articles. Reference lists of identified articles for inclusion were also hand-searched for suitable articles. Appendix 1 provides the search strategies employed for each database.

Study Selection

Studies were independently screened for inclusion by two reviewers, utilising a referential standardised proforma. Eligibility for study inclusion was defined as: a study of human patients with TBI, with the measurement of intracranial pressure via intraparenchymal or intraventricular pressure monitor, with insertion of an external lumbar drain for the purposes of CSF drainage with the therapeutic aim of ICP/CPP control.

Data Extraction

Data were extracted from included reports by two reviewers: conducted independently in duplicate, using piloted forms. Data extracted included study characteristics, patient demographics, number of patients, ICP measurement method and frequency, external lumbar drainage timing and drainage protocol, patient selection/exclusion criteria, ICP/CPP control outcome, avoidance of treatment intensity level escalation, and functional outcome.

Risk of Bias in Individual Studies

Two reviewers assessed the risk of bias of each individual study using the Risk Of Bias in Non-Randomised Studies Tool (ROBINS-I) [28], rating each level of bias as low, medium, serious, or critical. Unresolved discrepancies were discussed with a third party.

Synthesis of Results

The heterogeneous nature of data collection and analysis methods precludes our ability to combine and synthesise results. As such, a descriptive summary will be presented, with all included studies tabulated. Data are presented as given by the respective authors: data was not converted to a homogenous outcome reporting metric. Any missing summary statistics are identified by stating their absence in the tabular results presentation. Qualitative assessment of the confidence in the body evidence for outcomes was performed and presented as a narrative.

Results

Study Selection

**Figure 1:**
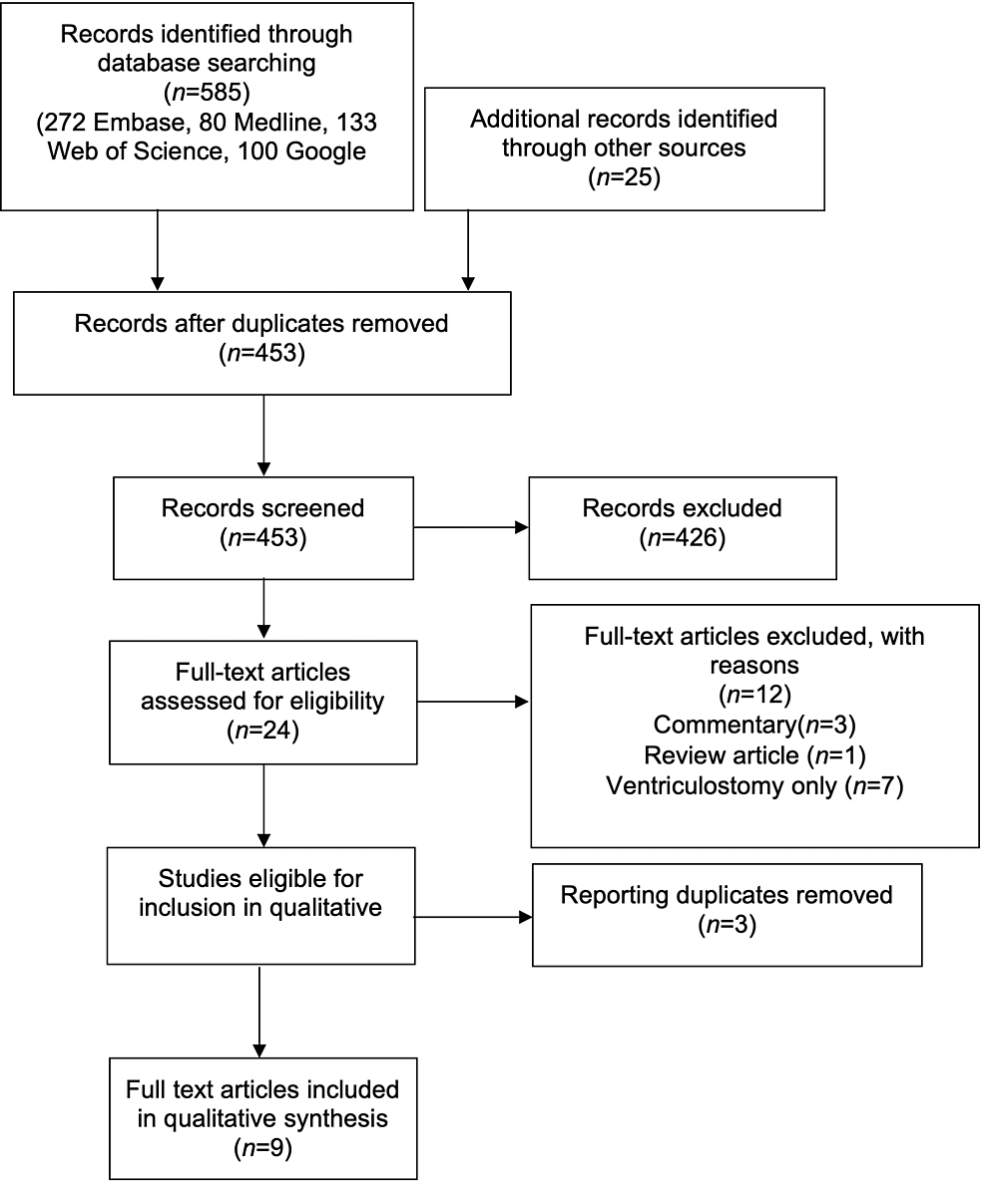
Search results systematic review flow diagram

A search of the literature pertaining to lumbar drainage in TBI yielded 610 results. After the removal of duplicates, 453 research items were screened against the inclusion criteria. Twenty-seven full text items were retrieved for eligibility assessment, of which 12 studies were considered to fulfil the criteria (Figure 1). Reasons for exclusion included commentary papers, review articles, and different procedures, further highlighted in the diagram.

Of the 12 research items eligible for inclusion [23,25,29-36,37], all were published articles. Three of these articles included data presented in previously published articles: Munch et al., 2001 data were later published in Tuettenberg et al., 2009; Murad et al., 2008 data were later published in Murad et al., 2012; and Baldwin et al., 1991 data were later published in Levy et al., 1995. The most recent publications were included in the full qualitative synthesis; prior publications were not included to avoid duplicate reporting of data.

Study and Patient Demographics

Three articles were presented as case series [29,32,34], with another presented as a case report and accompanying case series [37]. Five studies were presented as observational studies: three retrospective [31,33,35] and two prospective [23,25]. Three studies utilised a mixed cohort of patients with TBI and aneurysmal subarachnoid haemorrhage (SAH) and/or intracerebral haemorrhage (ICH) [23,25,35], with six studies including only patients with TBI [29,31-34,37]. One study included only paediatric patients [32], seven studies included only adult patients [25,29,31,33-35,37] and one included adults and children [23]. In total, 230 patients were included in the studies, of which 159 were patients with TBI. The demographics of included patients are reported in Table 1. Due to the manner in which the results were reported, the present authors were unable to delineate results pertaining only to TBI patients in the studies using a mixed cohort; as such the results will be presented together, noting where a mixed cohort is implicated where the results of these studies are discussed. Manet et al., 2016 [34] and Manet et al., 2017 [35] utilised a sub-cohort, including only patients with features of external hydrocephalus.

**Table 1:** Characteristics of patients in included studies *excluding missing data for one patient with unknown initial GCS TBI = traumatic brain injury, SAH = subarachnoid haemorrhage, ICH = intracerebral haemorrhage, EH = external hydrocephalus, GCS = Glasgow coma scale, EVD = external ventricular drain, ICP = intracranial pressure, CT = computed tomography

Authors	Cohort	n =	TBI n	Other n	Mean initial GCS	Mean age	Gender F:M	Inclusion criteria	Exclusion criteria
Abadal Centellas et al, 2007 [29]	Adults, TBI	17	17	0	8	32.5 ± 13.3	4:13	Refractory ICP after first and second level measures	CT criteria
Bauer et al, 2017 [31]	Adults, TBI	8	8	0	10.1*	54.1 (27 -70)	2:6	Refractory ICP with either small ventricles or high ICP disproportionate to CT	CT criteria
Levy et al, 1995 [32]	Children, TBI	16	16	0	6.2	Not stated	5:11	Considered if sustained ICP >25 on aggressive medical therapy	Nil stated
Llompart Pou et al, 2011 [33]	Adults, TBI	30	30	0	9	34.9±12.5	5:25	Refractory ICP after first and second level measures	CT criteria
Manet et al, 2016 [34]	Adults, TBI (EH)	4	4	0	10	53.5±7	2:2	Failure of maximal medical management and not suitable for or failed EVD, with increasing volume of CSF in SAS, paradoxical to ICHT	CT criteria, no mass lesion or CT factors indicating craniotomy or decompressive craniectomy
Manet et al, 2017 [35]	Adults, TBI/SAH/ICH (EH)	33	22	11 (SAH n=10, ICH n=1)	7.9	51 (median) (34-61)	Not available	External hydrocephalus from acute brain injury, refractory ICP with radiological evidence of external hydrocephalus	CT criteria
Murad et al, 2012 [25]	Adults, TBI/SAH	15	10	5 (SAH)	6.8	36.9 (19-60)	3:12	Aged 18-99 with ventriculostomy catheter, ICP >20mmHg no longer responsive to medical criteria	Unevacuated focal mass lesion, patients transferred with lumbar drains
Tuettenberg et al, 2009 [23]	Adults, TBI/SAH	100	45	55 (SAH)	7±4 (TBI 6.4±3)	43.7 (TBI 37.6±18.1)	Not available	Ongoing ICP >20, EVD in situ, CT parameters and acceptable clotting	Nil stated
Willemse, 1998 [37]	Adults	7	7	0	6	26 (21-35)	3:4	Lumbar drainage was only instituted in the absence of focal mass lesions and with discernible basilar cisterns on computerised tomography (CT) scan	Nil stated
Total		230	159	71					

Whilst our exclusion criteria resulted in the removal of studies which achieved CSF diversion through EVD alone, five of the nine studies included patients with an EVD in addition to ELD. In total, 100 patients received an ELD alone, and 130 patients received ELD + EVD. In three studies, all participants had both EVD and ELD [25,32,37], with EVD preceding ELD, two studies had a mixed cohort of ELD with and without EVD [23,35]. Llompart-Pou et al., 2011 [33] included two patients (of 30) who received an EVD though it is not clear whether the insertion of EVD preceded that of ELD. Patients in the remaining studies had ELD alone, with patients included in Manet et al., 2016 [34] only receiving ELD where EVD insertion had failed or was contraindicated. No study reported comparative outcome measures between groups with or without EVD. Furthermore, in such cases of ELD + EVD, there is variability of whether EVD was used for drainage, pressure monitoring, or both, with insufficient data reporting based on sub-group (ELD+EVD vs ELD) stratifications. As such, no conclusions may be drawn as to the relative potential advantages of implementing dual modality CSF drainage.

Eight studies only included patients with either: no surgical intracranial lesion or a surgical intracranial lesion post-evacuation; in one study, this was not specified [29]. All study protocols only instigated CSF diversion where medical management options had failed, all specifying at least hyperventilation and osmotic therapy in their ICP management protocols. Six studies indicated use of barbiturate coma [23,29,32-34,37] and five indicated use of hypothermia [29,33-35,37]. Four studies indicated the use of ventricular drainage prior to ELD [23,25,35,37] where possible.

Efficacy

All studies reported a marked reduction in ICP after CSF diversion with an ELD. Individual study results are reported in Table 2. The three studies [23,31,33] reporting the outcome of statistical tests of this effect all reported a statistically significant reduction in ICP after the introduction of lumbar drainage. Of four studies [23,25,29,32] presenting data or observations on the effect of lumbar drainage on CPP, all reported a positive effect. Four studies presented observations on the effect of lumbar drainage on treatment intensity or requirement for other ICP-lowering therapies [23,25,29,35]. All four reported a beneficial effect on the requirement of other therapies after the institution of lumbar drainage.

**Table 2:** Efficacy measures of ELD reported by included studies *denotes where no summary statistics are available TBI = traumatic brain injury, SAH = subarachnoid haemorrhage, ICH = intracerebral haemorrhage, EH = external hydrocephalus, EVD = external ventricular drain, ICP = intracranial pressure, CPP = cerebral perfusion pressure

Authors	Cohort	EVD?	ELD volume drained	Effect on ICP and summary statistics	CPP control outcomes	Prevention of TIL escalation?
Abadal Centellas et al., 2007 [29]	n =17 (adults, TBI)	No	Initial drainage of CSF to low ICP as described was 8.0 +/- 5.7 mL.	Mean ICP before and one hour after placement of ELD was 30.9 +/- 7.9 and 14.1 +/- 5.9 mm Hg. Excellent/good control of ICP achieved in 76% by day one and 94% by day three. *	Improvement in CPP in all patients	Excellent control of ICP (no mannitol or hypertonic saline used over 24hr period) in 94% day three post ELD
Bauer et al., 2017 [31]	n=8 (adults, TBI)	No	23.5 ml/24 h (mean, SD 16.41, range 0–40 ml)	Lumbar CSF removal led to a reduction of ICP in all patients. Mean ICP was 22.3 mmHg (SD 3.0) before CSF drainage and was 13.9 mmHg (SD 4.7) after drainage (p = 0.002).	Not documented	Not reported
Levy et al., 1995 [32]	n=16 (children, TBI)	Yes in all	Not stated	Fourteen of the 16 children had an abrupt and lasting decrease in ICP after placement of the lumbar drain, which obviated the need for continued aggressive medical therapy *	CPP improved in one case, otherwise not documented	Not reported
Llompart Pou et al., 2011 [33]	n=30 (adults, TBI)	No	Not stated	ICP before and one hour after ELD placement was 33.7±9.0 and 12.5±4.8 mmHg respectively, a decrease in ICP of 21.2±8.3 mmHg (p < 0.0001)	Not documented	Not reported
Manet et al., 2016 [34]	n= 4 (adults, TBI, EH)	No	Not stated	This procedure resulted in the immediate and long-lasting control of ICP: decrease from mean ICP of 37 ± 5 to mean 5 ± 2. *	Not documented	Not reported
Manet et al., 2017 [35]	n=33 (adults, TBI/SAH/ICH, EH)	Mixed (8 of 33)	Median CSF flow was 119 ml (96–280) per day	The ELD procedure led to a marked averaged reduction of ICP over the following 6 h by 16 mmHg (13–24), from 25 mmHg (20–31) before to 7 mmHg (3–10) after ELD (p < 0.001)	Not documented	Sedation was reduced in 25 patients (75%) within 24hr after ELD insertion
Murad et al., 2012 [25]	n=15 (adults, TBI/SAH)	Yes in all	Not stated	Reduced from mean of 28.2 mm Hg +/- 6.5 to 10.1 +/- 7.1 (p < 0.001). Requirements for hyperosmolar therapy, sedatives, paralytics decreased (p < 0.05)	CPP increased from 76.7 mmHg +/- 19.8 to 81.2 +/-10.2	Reduced patients requiring boluses of osmotic therapy from 12/15 to 1/15
Tuettenberg et al., 2009 [23]	n=100 (adults, TBI/SAH)	Mixed (84 of 100)	Not stated	Significant reduction in ICP from 32.7 ± 10.9 to 13.4 ± 5.9 mm Hg (p < 0.05)	Increase in CPP from 70.6 ± 18.2 to 86.2 ± 15.4 mm Hg (p < 0.05)	All modalities reduced except hyperventilation
Willemse, 1998 [37]	n=7 (adults, TBI)	Yes in all	Not stated	Five of the seven patients had a lasting decrease in ICP after lumbar drainage and survived.*	Not documented	N/A

Safety

Of 230 patients, there were 14 reports of either radiological or clinical signs of cerebral herniation after insertion of ELD (Table 3). One study [23] reported 12 of the 14 incidences of cerebral herniation, but the relationship between the development of clinical/radiological signs of cerebral herniation and the introduction of lumbar drainage is not described. The study did not present the relative incidence of these complications in TBI vs SAH patients, nor in ELD vs ELD + EVD patients. One of these episodes was reported as occurring due to the iatrogenic disconnection and uncontrolled drainage from the lumbar drain, and the patient survived without a neurological deficit. In the eight patients of these 12 who subsequently died, the authors did not attribute any death to, or as a complication of, lumbar drainage. There were two further incidences of herniation reported in the included literature, one patient exhibited only radiological signs [31] (bilateral uncal herniation), and one patient developed a fixed dilated pupil four hours after insertion of ELD which returned to normal after “an emergent craniectomy” and the patient made a “good neurological recovery” [25].

**Table 3:** Adverse events and complications reported in included studies TBI = traumatic brain injury, SAH = subarachnoid haemorrhage, ICH = intracerebral haemorrhage, EVD = external ventricular drain, ELD - external lumbar drain, LD = lumbar drainage, CSF = cerebrospinal fluid

Authors	Cohort	EVD?	CSF infections	LD revisions	Number requiring a shunt	Cerebral herniation post-ELD?	Details
Abadal Centellas et al., 2007 [29]	n=17 (adults, TBI)	No	0 (3 contaminants)	2 (5 blockages)	0	n=0	No pupil changes in 24 hours post insertion
Bauer et al., 2017 [31]	n=8 (adults, TBI)	No	None stated	None stated	None stated	n=1	One patient showed an uncal herniation on both sides after lumbar CSF withdrawal without mydriasis or other clinical signs of cerebral herniation
Levy et al., 1995 [32]	n=16 (children, TBI)	Yes in all	None stated	None stated	3	n=0	Two patients had fixed and dilated pupils prior to ELD insertion, remained so after lumbar drainage commenced
Llompart Pou et al., 2011 [33]	n=30 (adults, TBI)	No	1 (4 with positive cultures, 3 contaminant)	4 (8 obstruction)	3	n=0	No pupil changes in 48 hours post insertion
Manet et al., 2016 [34]	n=4 (adults, TBI)	No	0	0	0	n=0	N/A
Manet et al., 2017 [35]	n=33 (adults, TBI/SAH/ICH)	Mixed (8/33)	1	0	5	n=0	N/A
Murad et al., 2012 [25]	n=15 (adults, TBI/SAH)	Yes in all	0	None stated	None stated	n=1	Fixed dilated pupil four hours after ELD insertion, which returned to normal after surgery hours
Tuettenberg et al., 2009 [23]	n=100 (adults, TBI/SAH)	Mixed (84/100)	7 (all also had EVD)	14/100	None stated	n=12	No relationship to ELD described. All had unilateral mydriasis. 4/12 survived. Three patients died from cerebral herniation secondary to intracranial hypertension, three had a devastating injury prior to ELD, one died from pulmonary embolism, one died from cerebral infarction. No reports of morbidity or mortality directly from ELD
Willemse, 1998 [37]	n=7 (adults, TBI)	Yes in all	None stated	None stated	None stated	n=0	N/A

There were nine reported incidences of central nervous system (CNS) infection in the included studies. A further six patients had a positive cerebrospinal fluid culture sample which was treated as a contaminant. Seven out of nine CNS infection cases were reported to have both EVD and ELD in situ. There were 20 incidences of lumbar drain failure requiring revision. Eleven patients were reported to require long-term CSF diversion with the insertion of a CSF shunt.

Eight studies reported functional outcomes [23,29,31-35,37], and one reported mortality alone [25]. No study explicitly reported a case whereby a complication from the use of a lumbar drain contributed to mortality or poor neurological outcome.

CT Criteria

Seven of the nine studies stipulated the requirement of a CT head prior to proceeding with the insertion of an ELD [23,29,31,33-35,37]. Two studies required this in the preceding 24 hours [29,33] and one in the preceding eight hours [31]. All studies requiring CT described imaging criteria [23,29,31,33-35,37], all including discernible basal cisterns and the absence of a surgical mass/cerebral herniation. Three studies also included the requirement of a midline shift of <10mm [29,33,35]. Manet et al., 2017 [35] used the presence of “a gradual development of subdural or subarachnoid collections located in the Sylvian and/or interhemispheric fissures and/or cortical sulci” as evidence of external hydrocephalus and thus inclusion in their study. Bauer et al., 2017 [31] proposed and utilised a scoring system based on the patency of the prepontine and quadrigeminal cisterns and the absence of uncal and foraminal herniation on the pre-procedural imaging.

ELD Drainage Protocol

Eight studies utilised an ELD in all patients [23,25,29,32-35,37]. One study used a single lumbar puncture or intermittently open drainage in four of nine patients [31]. Of the eight studies which utilised only ELD, six used protocols with a continuous drainage strategy [23,29,32-35]. One study [25] used varying methods, either continuous pressure-controlled drainage or fixed drainage at 10 ml/hr. One did not state their drainage protocol [37]. Four studies describe an initial high drainage protocol until ICP control was achieved, with intensive pupillary examination [23,32,34,35], two of which were in external hydrocephalus. Further details of specific drainage protocols are available in Appendix 2.

Heterogeneity of Studies

A descriptive review methodology was utilised due to the considerable heterogeneity of included studies. Practice variation in CSF diversion in TBI is considerable, relying on physician preference, availability of resources and differing guideline approaches for implementation of CSF diversion based on variable pre-defined ICP thresholds. Secondly, significant variation in of outcome measures in the included studies was identified. These included the modified Rankin scale, ICP metrics of efficacy (gross ICP or ICP change), and the Glasgow Outcome Scale. Similarly, complication reporting was variable in its detail and objectivity. Given such heterogeneity in both implementation of the intervention and in reporting of outcome measures, it was not deemed valuable or reliable to perform quantitative synthesis.

Risk of Bias Assessment

The risk of bias across was deemed low or moderate in all domains across all studies. Principle sources of moderate bias identified across studies were limited consideration of the possibility of confounding factors, limited consideration of selection bias, and classification of intervention. Overall, of the nine studies, all were deemed to have a moderate risk of bias (Appendix 3).

Discussion

The aim of this systematic review was to evaluate the available efficacy and safety data regarding the use of lumbar drainage for ICP control in TBI. The literature identified represents a small evidence base supporting the efficacy of lumbar drainage, where nine independent studies observed the effects in 159 patients with TBI (230 patients overall). Other than infection, need for drain revision, and transient mydriasis, one incident of morbidity (not affecting neurological outcome) was reported in these studies; though this is suggestive of the safety of ELD in TBI, the small cohort is insufficient to conclusively confirm it.

Efficacy

The data presented in the included studies supports the efficacy of lumbar drainage for ICP control as an adjunct to best medical therapy where optimal medical management has failed. Every study reported a beneficial effect for ICP control overall, with all of those performing statistical analysis finding a significant reduction in ICP following ELD insertion. Studies reporting mean reduction values utilised data one hour before and after commencing lumbar drainage, which unanimously confirmed an immediate effect. The positive effects on reducing the need for medical interventions such as osmotic therapy are also encouraging and were observed in all four studies reporting this outcome. The effect has been observed, though only reported for small numbers of patients, with increasing efficacy up to three days after the instigation of lumbar drainage [29]. Together with one-hour pre- and post-ELD ICP readings, the effect of lumbar drainage appears to be both immediate and sustained.

A significant reduction in ICP was reported in four studies which included ventriculostomy drainage prior to lumbar drainage [23,25,35,37], suggesting that ELD offers both a benefit when used as the sole modality of CSF diversion and an additional benefit when used in combination with EVD.

Functional outcomes were not considered as a marker of “efficacy”; rather, ICP control can be considered as a surrogate marker of potentially improving functional outcomes due to the association of intracranial hypertension and poor outcome [38]. The numbers enrolled in these combined studies are by far insufficiently powered to assess the effect on functional outcome.

Safety

The available data identified by this systematic review is insufficient to draw firm conclusions on the safety profile of ELD in the management of raised ICP in TBI; however, the included studies here have not reported incidents of the usage of ELD directly contributing to mortality. A single case of ELD usage has been reported to result in pupillary changes requiring urgent surgery with good neurological recovery). However, the absence of significant complications in a series of 159 patients with TBI is unlikely to generate sufficient confidence to assuage the reservations held by some neurosurgical centres. In the single reported case of cerebral herniation requiring surgery, this was urgently recognised and appropriately managed, resulting in good neurological recovery. Despite historical concerns of “coning” from lumbar puncture or drainage, the results suggest that in the modern intensive care unit, there is sufficient clinical monitoring to facilitate early recognition of cerebral herniation through clinical and radiological assessment and prompt remedial action.

Whilst a high mortality rate is not justification for therapeutics with the potential for harm, the alternative to lumbar drain is typically insertion of EVD; where this fails, decompressive craniectomy is typically indicated, and neither can be considered to be low morbidity. Whilst not robust, the results here are suggestive of a low to very low risk of severe morbidity or mortality as a result of ELD in selected patients. When considering surgical risk, ELD offers the potential benefit of achieving ICP control without the need for EVD or decompressive craniectomy.

Infection rates of the included studies were 3.9%, though some incidences of infection were in patients with both EVD and ELD. This incidence is similar to that reported in the literature, with infection rates of ELD reported as 4.2-7% [39,40] and EVD reported as 7-9% [39,41].

CT Criteria

Given the concern of obliterated basal cisterns preventing CSF movement across the foramen magnum, considered patient selection would be a necessary component of implementing lumbar drainage. Patient selection on the basis of radiological criteria was common in most of the included studies. A number of the included studies refer to the protocol in Munch et al. 2001 to cite the earliest example, though Willemse et al. 1998 include a similar protocol: discernible basal cisterns and absence of surgical mass lesions. The absence of tonsillar herniation, midline shift of less than 10 mm, and patency of prepontine and interpeduncular cisterns have been more recent additions. Due to the low complication rate, it would be difficult to identify which protocol is optimal for safety in patient selection. Further details of the radiological features of those with transient mydriasis are not available to make a post-hoc assessment of which further features transpired to be associated with risk.

Drainage Strategies

Drainage strategies varied amongst studies, with insufficient data reporting to draw conclusions regarding the optimal protocol. The protocols where described adopt broadly similar strategies: greater drainage permitted to achieve ICP control (<20 mmHg), with subsequent “maintenance” drainage (ICP in the region of 10-20 mmHg) and closure of the drain where ICP was low (<10 mmHg). Pressure settings of the ELD varied widely between 0 cm above the foramen of Monro (FOM) and 20 cm above. The study reporting a fixed dilated pupil four hours after ELD insertion allowed free drainage at 0 cm [25], a strategy which physiologically would permit continued drainage in the context of early cerebral herniation. Similarly, Tuettenberg et al., observing 12 episodes of unilateral mydriasis in their mixed TBI/SAH cohort, permitted free drainage at 5 cm above the FOM. Differing strategies for drainage should also consider nursing practicalities: continuous drainage at lower pressure settings is more likely to result in high volume outputs between nursing monitoring intervals.

When considering the physical and biomechanical aspects of drainage, a minimum volume is required to produce an acceptable ICP/CPP for the purpose of optimising perfusion and minimising the requirement for intervention. Therefore a sliding scale hourly drainage target, with an arbitrary maximum per hour of 10 ml, may provide an attractive and safe starting point in the views of the authors. Physically the volume of any potentially herniated neuro-axial tissue cannot exceed that of the volume of fluid drained, supporting further the likely safety of small incremental drainage as opposed to open pressure regulated drainage where theoretically no limit on the volume of drained fluid or herniating tissue exists.

Concomitant EVD

As outlined above, ELD appears to offer additional benefits when used as an adjunct to EVD in ICP control. In the largest included study [23], 84/100 patients had received ventricular drainage prior to ELD which had failed to control their ICP, though it is not specified in the methods whether ventricular drainage continued once ELD was inserted. Given that the concerns surrounding lumbar drainage are centred on a pressure gradient across the foramen magnum, simultaneous EVD and ELD drainage theoretically reduces the risk of this occurring. However, this study reported a relatively high rate of signs of cerebral herniation (with unclear timing in relation to ELD).

Comparison of CSF Diversion Techniques

One important consideration is the relative complication rate of the two strategies: EVD and ELD. A systematic review [42] found the main (early) EVD-related complications included: infection (12.8%), haemorrhage (1.5%), device failure (14.9%), and malposition (10.1%). This review focused on the timing of intervention and identified that early EVD intervention correlated with a higher infection rate (12.8%) than late (8.3%). Indirect comparison with ELD complication rates identified in this study demonstrates broadly similar rates. Included studies reported infection associated with ELD (5.7%) and device blockage (17.0%) complications. Three included studies did not report any complications from ELD use in their cohorts [20,22,37]. Given the predominance of retrospective study designs, underreporting of device-associated complications is likely, and the risk of reporting and observation is high.

The prospect of utilising ELD instead of, or as an adjunct to, EVD in selected patients has some potential benefits. The prevalence of “slit” ventricles in the TBI cohort with intracranial hypertension presents a technical challenge. The principal consideration of the use of EVD or ELD in the context of TBI is overall safety. The included studies have demonstrated that, in particular, in patients with stringently controlled drainage and monitoring parameters, ELD can be implemented safely in this context. However, further prospective study is required to be able to draw valid conclusions on the relative risks and merits of the two available modalities.

Limitations

The limitations of the present study are primarily due to the heterogeneity of both the included studies and the data that they have presented. Exclusion of studies with a mixed cohort of brain injury aetiology and adult/paediatric cohort would have further narrowed the low patient and study numbers available for consideration by this study. As such, we have included these, particularly given the necessity to be comprehensive when considering the intervention’s safety profile. Further to this, even with these inclusions, the numbers of patients and studies are low, though identifying paucity in the literature serves as an outcome of this review. The results should be interpreted with caution in recognition that the available literature is a severe limitation of this study. However, this serves to highlight the necessity for high-quality evidence through future studies. The detail in reporting the potential complication events was variable but generally poor. This is, in part, likely to be related to the incidence of clinical and radiological evidence of “coning” being a potential part of the natural history of severe TBI with refractory intracranial hypertension. As such, we have only considered complications to be related to lumbar drainage, where the respective authors have themselves reported these events to be a consequence of lumbar drainage. Given the heterogeneity of cohorts and data reporting, it was not deemed suitable nor beneficial to perform a meta-analysis, and a narrative review of the data is inherently limited in the conclusions that it may draw.

## Conclusions

The literature appears to support the efficacy of lumbar drainage in ICP control where medical management has failed, though the data on its safety is encouraging but insufficient to draw conclusive recommendations. The efficacy and safety profile of ELD in comparison to EVD is not known, though ELD appears to be beneficial for ICP control both alone and where EVD has failed. Although the evidence base is insufficient to draw firm conclusions, based on the available evidence, there is no clear indication that the complication rates of ELD are greater than those of EVD. Further large prospective observational studies are required to generate sufficient support for an acceptable safety profile, with the possibility of subsequent randomised controlled studies to ultimately assess therapeutic parity.

## Appendices

Appendix 1

The following search strategies were employed from these databases:

Embase

1. exp brain injury/

2. exp drainage/ or exp cerebrospinal fluid drainage/

3. exp intracranial hypertension/

4. 1 and 2 and 3

Medline

1. exp brain injury/

2. exp intracranial hypertension/

3. exp Drainage/ or drainage.mp

4. 1 and 2 and 3

Cochrane

1. brain injury AND intracranial hypertension AND drainage

Nice

1. brain injury AND intracranial hypertension AND drainage

Google Scholar

“brain injury intracranial hypertension drainage”

Appendix 2

**Table 4:** Table of included studies with detail of modality and protocol of lumbar drainage TBI = traumatic brain injury, SAH = subarachnoid haemorrhage, ICH = intracerebral haemorrhage, ICP = intracranial pressure, FOM = foramen of Monro

Authors	Cohort	Method	Continuous or Intermittent	Level of drain	Drainage protocol
Abadal Centellas et al., 2007 [29]	n=17 (adults, TBI)	Lumbar drain	Continuous	FOM	Drainage of cerebrospinal fluid (CSF) was done continuously when ICP raised over 20 mm Hg. If ICP dropped below 10 mm Hg, CSF drainage was immediately stopped. When ICP increased to 15 mm Hg, the drainage system was opened again
Bauer et al., 2017 [31]	n=8 (adults, TBI)	Drain or puncture	Mixed	FOM	Lumbar drain with continuous drainage (n=5) or single LP or intermittently open drainage (n=4)
Levy et al., 1995 [32]	n=16 (children, TBI)	Lumbar drain	Continuous	Head	5-15 cm above the head depending on ICP, gradual decompression over 1-3 minutes
Llompart Pou et al., 2011 [33]	n=30 (adults, TBI)	Lumbar drain	Continuous	10-15cm above FOM	When the ICP was over 20 mmHg, CSF was drained continuously with hourly recordings. After the ICP decreased below 10 mmHg, the external lumbar drain was closed, CSF removal stopped and the patient’s head was placed at 0 degrees to minimise the risk of cerebral herniation. When ICP increased above 15 mmHg the system was opened and the patient’s head placed at 30 degrees again to reinitiate removal
Manet et al., 2016 [34]	n=4 (adults, TBI)	Lumbar drain	Continuous	Tragus	A careful initial CSF withdrawal was achieved at a slow rate (mL by mL), in the presence of the attending neurosurgeon and intensivist, with a continuous papillary examination. When ICP acceptable, CSF drainage was fixed 20 cm above the tragus to maintain safe, continuous CSF drainage
Manet et al., 2017 [35]	n=33 (adults, TBI/SAH/ICH)	Lumbar drain	Continuous	10-15 cm above EAM	Initially withdrawn at 1ml/min with pupillary examination until CPP target (60-70) reached. Then set at 10-15 cm above a zero reference set at the level of the foramen of Monro (external acoustic meatus) and adjusted to maintain a continuous flow of drainage, i.e. 5-15 ml/h
Murad et al., 2012 [25]	n=15 (adults, TBI/SAH)	Lumbar drainage	Varied	0 above FOM	Free drainage in some, 10ml per hour in others (no difference between the two groups)
Tuettenberg et al., 2009 [23]	n=100 (adults, TBI/SAH)	Lumbar drainage	Continuous	5cm above FOM	5-20ml CSF carefully aspirated then free drainage until ICP 10-15 regardless of volume
Willemse, 1998 [37]	Adults	Lumbar drainage	Not stated	5-15 cm above FOM	Not stated

Appendix 3

**Table 5:** Risk of bias assessment of the included studies, using the ROBINS-I tool ROBINS-I = Risk Of Bias in Non-Randomised Studies Tool

Authors	Study Design	Confounding	Selection participant	Classification of intervention	Missing data	Outcome measurement	Selection of reported result	Overall risk of bias
Abadal Centellas et al., 2007 [29]	Retrospective	Moderate	Moderate	Moderate	Low	Low	Low	Moderate
Bauer et al., 2017 [31]	Retrospective	Moderate	Moderate	Moderate	Low	Low	Moderate	Moderate
Levy et al., 1995 [32]	Retrospective	Moderate	Low	Moderate	Moderate	Low	Low	Moderate
Llompart Pou et al., 2011 [33]	Retrospective	Moderate	Moderate	Low	Moderate	Moderate	Low	Moderate
Manet et al., 2016 [34]	Retrospective	Moderate	Moderate	Low	Low	Low	Low	Moderate
Manet et al., 2017 [35]	Retrospective	Moderate	Low	Low	Moderate	Moderate	Low	Moderate
Murad et al., 2012 [25]	Prospective	Moderate	Low	Moderate	Low	Low	Low	Moderate
Tuettenberg et al., 2009 [23]	Prospective	Moderate	Moderate	Moderate	Moderate	Low	Low	Moderate
Willemse, 1998 [37]	Retrospective	Moderate	Moderate	Moderate	Moderate	Low	Low	Moderate

## References

[1] Carney N, Totten AM, O'Reilly C (2017). Guidelines for the management of severe traumatic brain injury, fourth edition. Neurosurgery.

[2] (1783). Observations on the structure and functions of the nervous system, illustrated with tables. Lond Med J.

[3] Kellie G (1824). An account of the appearances observed in the dissection of two of the three individuals presumed to have perished in the storm of the 3rd, and whose bodies were discovered in the vicinity of Leith on the morning of the 4th November 1821 with some reflections on the pathology of the brain: part I. Transac Med Chir Soc Edinburgh.

[4] Nwachuku EL, Puccio AM, Fetzick A, Scruggs B, Chang YF, Shutter LA, Okonkwo DO (2014). Intermittent versus continuous cerebrospinal fluid drainage management in adult severe traumatic brain injury: assessment of intracranial pressure burden. Neurocrit Care.

[5] Bales JW, Bonow RH, Buckley RT, Barber J, Temkin N, Chesnut RM (2019). Primary external ventricular drainage catheter versus intraparenchymal ICP monitoring: outcome analysis. Neurocrit Care.

[6] Chowdhury YA, Stevens AR, Soon WC (2022). Cerebrospinal fluid diversion for refractory intracranial hypertension: a United Kingdom and Ireland survey on practice variation. Cureus.

[7] Shore PM, Thomas NJ, Clark RS (2004). Continuous versus intermittent cerebrospinal fluid drainage after severe traumatic brain injury in children: effect on biochemical markers. J Neurotrauma.

[8] Griesdale DE, McEwen J, Kurth T, Chittock DR (2010). External ventricular drains and mortality in patients with severe traumatic brain injury. Can J Neurol Sci.

[9] Vourc’h G (1963). Continuous cerebrospinal fluid drainage by indwelling spinal catheter. Br J Anaesth.

[10] Basauri LT, Concha-Julio E, Selman JM, Cubillos P, Rufs J (1999). Cerebrospinal fluid spinal lumbar drainage: indications, technical tips, and pitfalls. Crit Rev Neurosurg.

[11] Bell RB, Dierks EJ, Homer L, Potter BE (2004). Management of cerebrospinal fluid leak associated with craniomaxillofacial trauma. J Oral Maxillofac Surg.

[12] Albu S, Florian IS, Bolboaca SD (2016). The benefit of early lumbar drain insertion in reducing the length of CSF leak in traumatic rhinorrhea. Clin Neurol Neurosurg.

[13] El Ahmadieh TY, Wu EM, Kafka B (2019). Lumbar drain trial outcomes of normal pressure hydrocephalus: a single-center experience of 254 patients. J Neurosurg.

[14] Guo X, Zhu Y, Hong Y (2020). Efficacy and safety of intraoperative lumbar drain in endoscopic skull base tumor resection: a meta-analysis. Front Oncol.

[15] Tan J, Song R, Huan R, Huang N, Chen J (2020). Intraoperative lumbar drainage can prevent cerebrospinal fluid leakage during transsphenoidal surgery for pituitary adenomas: a systematic review and meta-analysis. BMC Neurol.

[16] Fedorow CA, Moon MC, Mutch WA, Grocott HP (2010). Lumbar cerebrospinal fluid drainage for thoracoabdominal aortic surgery: rationale and practical considerations for management. Anesth Analg.

[17] Nash C (1937). Cerebellar herniation as a cause of death. Ann Otol Rhinol Laryngol.

[18] Masson C (1929). The dangers of diagnostic lumbar puncture in increased intracranial pressure due to brain tumor, with a review of 200 cases in which lumbar puncture was done. Arch NeurPsych.

[19] Duffy GP (1969). Lumbar puncture in the presence of raised intracranial pressure. Br Med J.

[20] Hepburn HH (1938). The risk of spinal puncture. Can Med Assoc J.

[21] Abulhasan YB, Al-Jehani H, Valiquette MA (2013). Lumbar drainage for the treatment of severe bacterial meningitis. Neurocrit Care.

[22] Zhang Q, Li H, Zhang K (2019). Lumbar drainage for the treatment of refractory intracranial hypertension in HIV-negative cryptococcal meningitis. Future Microbiol.

[23] Tuettenberg J, Czabanka M, Horn P (2009). Clinical evaluation of the safety and efficacy of lumbar cerebrospinal fluid drainage for the treatment of refractory increased intracranial pressure. J Neurosurg.

[24] Murad A, Ghostine S, Colohan AR (2008). Controlled lumbar drainage in medically refractory increased intracranial pressure. A safe and effective treatment. Acta Neurochir Suppl.

[25] Murad A, Ghostine S, Colohan AR (2012). A case for further investigating the use of controlled lumbar cerebrospinal fluid drainage for the control of intracranial pressure. World Neurosurg.

[26] Hawryluk GW, Aguilera S, Buki A (2019). A management algorithm for patients with intracranial pressure monitoring: the Seattle International Severe Traumatic Brain Injury Consensus Conference (SIBICC). Intensive Care Med.

[27] Moher D, Liberati A, Tetzlaff J, Altman DG (2009). Preferred reporting items for systematic reviews and meta-analyses: the PRISMA statement. BMJ.

[28] Sterne JA, Hernán MA, Reeves BC (2016). ROBINS-I: a tool for assessing risk of bias in non-randomised studies of interventions. BMJ.

[29] Abadal-Centellas JM, Llompart-Pou JA, Homar-Ramírez J, Pérez-Bárcena J, Rosselló-Ferrer A, Ibáñez-Juvé J (2007). Neurologic outcome of posttraumatic refractory intracranial hypertension treated with external lumbar drainage. J Trauma.

[30] Baldwin HZ, Rekate HL (1991). Preliminary experience with controlled external lumbar drainage in diffuse pediatric head injury. Pediatr Neurosurg.

[31] Bauer M, Sohm F, Thomé C, Ortler M (2017). Refractory intracranial hypertension in traumatic brain injury: proposal for a novel score to assess the safety of lumbar cerebrospinal fluid drainage. Surg Neurol Int.

[32] Levy DI, Rekate HL, Cherny WB, Manwaring K, Moss SD, Baldwin HZ (1995). Controlled lumbar drainage in pediatric head injury. J Neurosurg.

[33] Llompart-Pou JA, Abadal JM, Pérez-Bárcena J (2011). Long-term follow-up of patients with post-traumatic refractory high intracranial pressure treated with lumbar drainage. Anaesth Intensive Care.

[34] Manet R, Schmidt EA, Vassal F, Charier D, Gergelé L (2016). CSF lumbar drainage: a safe surgical option in refractory intracranial hypertension associated with acute posttraumatic external hydrocephalus. Acta Neurochir Suppl.

[35] Manet R, Payen JF, Guerin R, Martinez O, Hautefeuille S, Francony G, Gergelé L (2017). Using external lumbar CSF drainage to treat communicating external hydrocephalus in adult patients after acute traumatic or non-traumatic brain injury. Acta Neurochir (Wien).

[36] Münch EC, Bauhuf C, Horn P, Roth HR, Schmiedek P, Vajkoczy P (2001). Therapy of malignant intracranial hypertension by controlled lumbar cerebrospinal fluid drainage. Crit Care Med.

[37] Willemse RB, Egeler-Peerdeman SM (1998). External lumbar drainage in uncontrollable intracranial pressure in adults with severe head injury: a report of 7 cases. Acta Neurochir Suppl.

[38] Åkerlund CA, Donnelly J, Zeiler FA (2020). Impact of duration and magnitude of raised intracranial pressure on outcome after severe traumatic brain injury: a CENTER-TBI high-resolution group study. PLoS One.

[39] Schade RP, Schinkel J, Visser LG, Van Dijk JM, Voormolen JH, Kuijper EJ (2005). Bacterial meningitis caused by the use of ventricular or lumbar cerebrospinal fluid catheters. J Neurosurg.

[40] Coplin WM, Avellino AM, Kim DK, Winn HR, Grady MS (1999). Bacterial meningitis associated with lumbar drains: a retrospective cohort study. J Neurol Neurosurg Psychiatry.

[41] Bota DP, Lefranc F, Vilallobos HR, Brimioulle S, Vincent JL (2005). Ventriculostomy-related infections in critically ill patients: a 6-year experience. J Neurosurg.

[42] Chau CY, Mediratta S, McKie MA (2020). Optimal timing of external ventricular drainage after severe traumatic brain injury: a systematic review. J Clin Med.

